# Increasing freshwater supply to sustainably address global water security at scale

**DOI:** 10.1038/s41598-022-24314-2

**Published:** 2022-12-06

**Authors:** Afeefa Rahman, Praveen Kumar, Francina Dominguez

**Affiliations:** 1 Department of Civil and Environmental Engineering, University of Illinois at Urbana-Champaign, Champaign, IL USA; 2 Department of Atmospheric Sciences, University of Illinois at Urbana-Champaign, Champaign, IL USA; 3 Prairie Research Institute, University of Illinois at Urbana-Champaign, Champaign, IL USA

**Keywords:** Hydrology, Hydrology

## Abstract

While significant parts of the globe are already facing significant freshwater scarcity, the need for more freshwater is projected to increase in order to sustain the increasing global population and economic growth, and adapt to climate change. Current approaches for addressing this challenge, which has the potential to result in catastrophic outcomes for consumptive needs and economic growth, rely on increasing the efficient use of existing resources. However, the availability of freshwater resources is rapidly declining due to over-exploitation and climate change and, therefore, is unlikely to sustainably address future needs, which requires a rethink of our solutions and associated investments. Here we present a bold departure from existing approaches by establishing the viability of significantly increasing freshwater through the capture of humid air over oceans. We show that the atmosphere above the oceans proximal to the land can yield substantial freshwater, sufficient to support large population centers across the globe, using appropriately engineered structures. Due to the practically limitless supply of water vapor from the oceans, this approach is sustainable under climate change and can transform our ability to address present and future water security concerns. This approach is envisioned to be transformative in establishing a mechanism for sustainably providing freshwater security to the present and future generations that is economically viable.

## Introduction

Lack of adequate access to fresh water across vast regions of the globe^1,2^ poses a grand challenge for our time that needs a bold and immediate solution. Current approaches to addressing this challenge primarily by reducing and managing demand are proving inadequate^3^ as population and economic growth quickly absorb any capacity that is created through these measures^4–7^. Recycling and reuse of water have had noticeable success^8^ but inherently have limited scalability because they are fundamentally constrained by the available supply^9,10^. Effective solutions to increase the supply are at present limited, or they are practically non-existent since all resources are being exploited beyond sustainable capacity or rapidly dwindling due to climate change. For example, groundwater is being extracted far beyond renewable rates, and the groundwater table is falling at alarming rates in regions where freshwater is needed most^11^. Snowpack and glaciers that serve as water towers are thinning or receding under climate change, with snowmelt occurring earlier in the spring season than before^12^. Regions that are already water limited are becoming more so as climate-driven changes are creating further scarcity through reduced precipitation, increased evaporation, or both^13^. Options for fulfilling this increased need through transport from distant areas are also becoming increasingly less viable due to a decrease in water availability in the source regions. The southwestern United States is a compelling example of these challenges where the water level in the Colorado River reservoirs has been reducing during the past decade, reaching critically low levels during the summer of 2021, threatening both the water resources and power systems^14,15^. Many examples of such cascading influences exist around the globe^16–19^.

A potential solution for radically increasing freshwater supply is to tap into the practically limitless oceanic sources. While desalination offers such a technology, it has met with significant environmental concerns and, as a result, hasn’t been adopted as a scalable solution for addressing global water security concerns, although its role in meeting the needs of a large population in several critical regions of the globe cannot be understated^20,21^. Desalination is not only energy intensive; it also creates concentrated brine and other byproducts that create significant environmental challenges with the cost of disposal^22,23^. Here we establish the feasibility of an alternate approach to tap into the oceanic sources of water in an economically viable and environmentally friendly manner. We show that the proposed method is scalable in that it can be implemented to meet the needs of an arbitrarily large population, and it is practical under future climate scenarios in that it makes more water available in a warmer climate, thereby providing an important tool for developing resilience to climate change.

**Figure 1:**
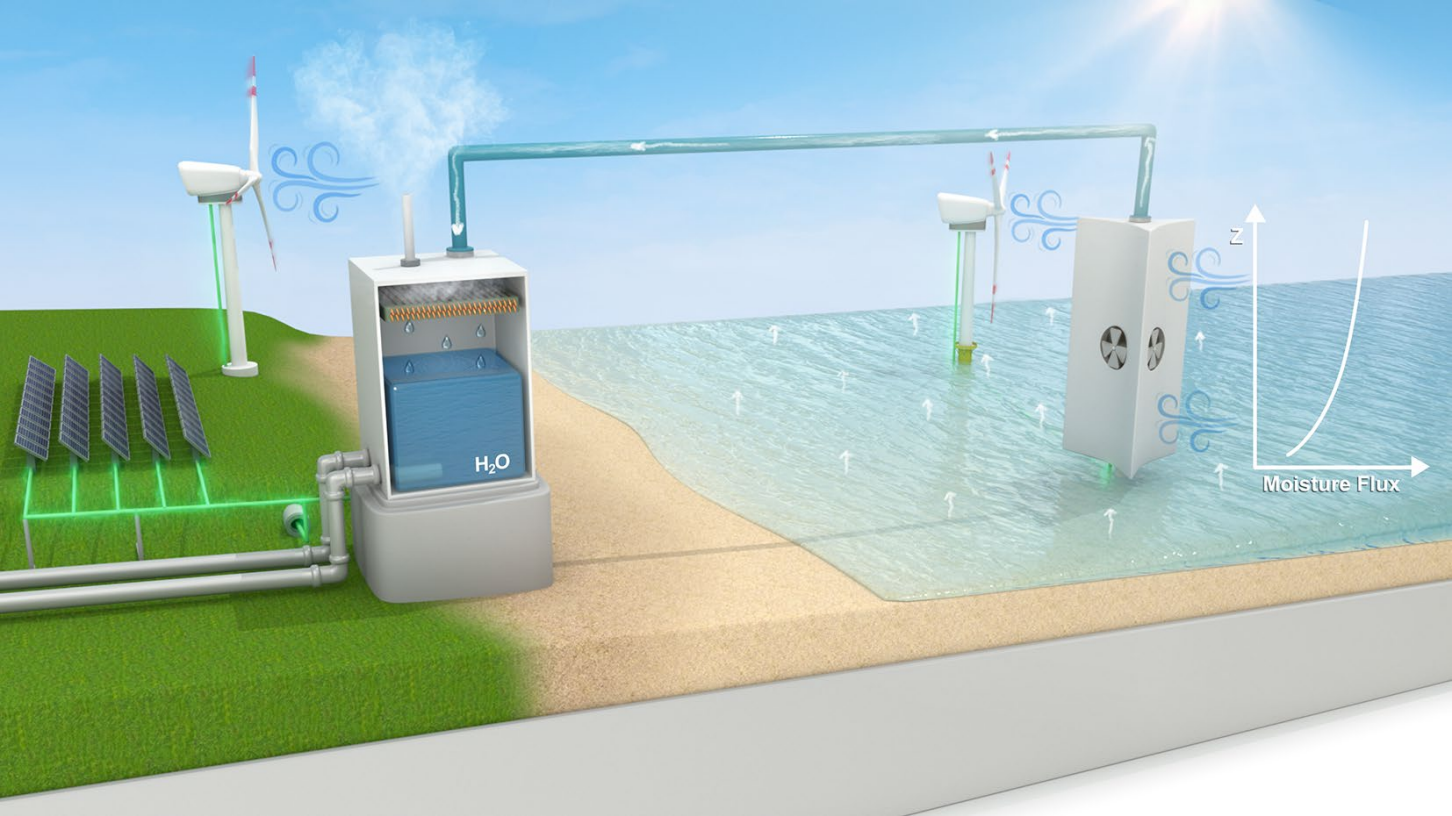
Schematic illustration of our proposed approach for capturing moisture above the ocean surface and transporting it to proximal land for improving water security. Evaporated moisture flux in the form of water vapor in the atmosphere above the ocean can be captured through an appropriately designed intake as conceptualized on the right, transported in the vapor phase through a conduit, and condensed over land as illustrated on the left to obtain fresh water. The moisture flux increases gently with altitude due to higher horizontal wind, offering the opportunity to design vertical capture surfaces. The intake can be designed to optimize the moisture intake taking into consideration the prevailing wind direction and its variation with altitude and time. A compressor can increase the efficiency of collecting the humid airmass at the intake. As illustrated in the Figure, renewable energy (wind or solar) can power the intake, transport, and condensation of moist air. We expect the intake to be located several kilometers offshore to ensure sufficient open water fetch isotropically around the intake. [Figure created using Adobe Photoshop, not to scale].

Our proposed approach consists of capturing water vapor from the atmosphere just above the ocean surface and transporting the moisture-laden air to proximal land where its condensation can provide fresh water (Fig. 1). The near-surface environments above the ocean have high humidity, whose daily and seasonal variations are primarily driven by the temperature of the oceanic surface and that of the air above. The former determines the evaporative capacity from the ocean, while the latter determines the saturated moisture holding capacity of the atmosphere. Variations in these temperatures, and hence the humidity in the atmosphere, are largely determined by the variation of solar radiation and wind velocities. The objective of this paper is to show that for water-stressed areas of the globe that are proximal to oceans, the availability of moisture in the near-surface atmospheric column not only makes significant generation of freshwater supply viable but offers a scalable approach for addressing water security challenges. Since this moisture in the atmosphere results from the natural evaporation of oceanic water, no environmentally harmful byproducts are expected. In essence, our approach mimics the natural physical process of the hydrologic cycle by which evaporated moisture from the ocean gets transported inland, cools, and condenses to then fall on the land surface as precipitation, except that we propose to engineer the pathway through which the evaporated moisture moves thus controlling the location of where the water is made available through controlled condensation.

We demonstrate the viability of this proposed approach by computing the quantity of extractable moisture that is available in the near-surface atmospheric column above the ocean. We then show that a vertical “capture surface” that is 210 m wide and 100 m tall, roughly corresponding to the vertically projected area of a large cruise ship, can provide a sufficient volume of extractable moisture to meet the daily potable needs of approximately 500,000 people, on average. These dimensions are chosen arbitrarily and implemented here only as a way to illustrate that the potential volume of water extracted can be significant. We expect that the actual implementation will encompass a significant variation of these dimensions based on prevailing local conditions and driven by needs and associated cost-benefit analyses. Our goal here is only to establish that a sufficiently large volume of moisture can be obtained through the proposed approach under the prevailing conditions, which is sufficient to meet the water demand of a large population. We then examine how this capacity may be impacted by climate change. This is important because an investment in such infrastructure will serve the population for decades, and we aim to ensure that its capacity will not degrade over time. Since such infrastructure is yet to be built, we also provide some thoughts on the cost structure to build and operate such facilities so they are competitive with existing operational desalination plants. Since we face severe shortages of freshwater in significant parts of the globe, our goal is that the option proposed here will primarily serve to augment existing capacities sustainably, but in some cases, they may serve to disengage unsustainable practices.

## Results and discussions

### Available moisture in atmosphere above oceans

We first compute the quantity of water available in an atmospheric column as the integrated water vapor flowing through a vertical column in the surface sublayer of the atmosphere of height *h* that is 1 m wide at a given location. Due to the non-linearity of its variation through the vertical column, this is computed as the sum of moisture fluxes through discrete horizontal layers, as illustrated in Figure S1. The mean moisture flux (MF, kg/m^2^s or equivalently liters/m^2^s), which is the rate at which the mass of water in the vapor phase moves horizontally per unit vertical area per unit time, is calculated as the product of the mean horizontal wind (*U*), the specific humidity (*q*), and the air density ($\rho _a$, assumed as 1.12 kg/m^3^^24^). Due to the influence of surface roughness, wind speed is lowest near the surface and increases with altitude. The specific humidity is highest near the surface, which serves as the moisture source, and decreases with altitude. The net effect is that the moisture flux in the atmosphere generally increases with altitude as the higher wind speed overcomes the reduction in humidity with altitude (Figure S1). This can be scaled linearly in the horizontal for any width, *w* (210 m in our illustration), to provide a reasonable estimate of moisture flux for the considered height *h*.

**Figure 2:**
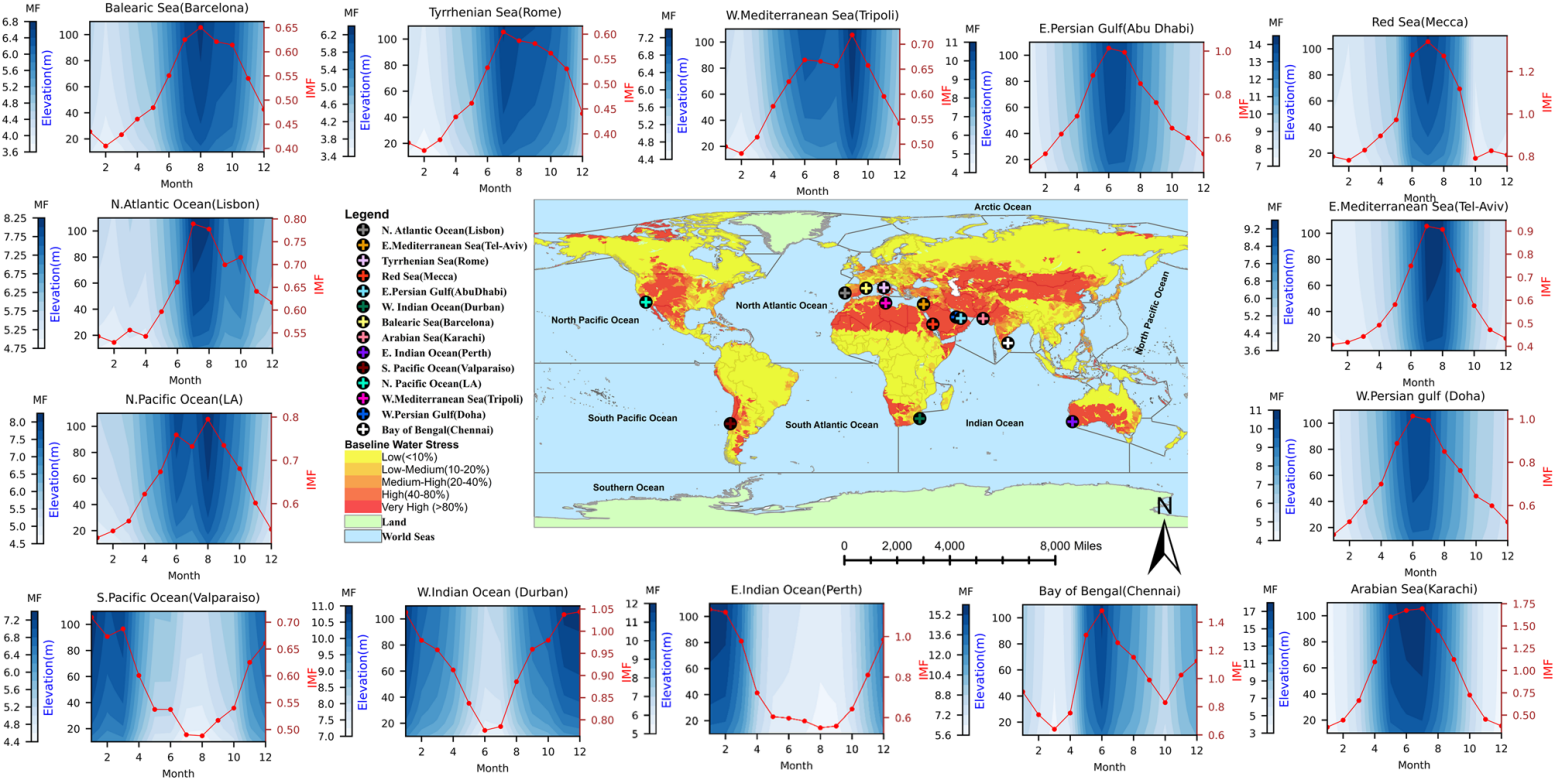
The location of the 14 study sites over the ocean closest to a dominant population center is depicted on a map of water stress (center). The variation of moisture flux through an atmospheric column from 10 to 110 m above mean sea level is also shown for each of the locations. The contour plots and line graph illustrate the change in daily moisture flux as a function of height and the monthly distribution of available water vapor, respectively. For each location, monthly averages (in red) of moisture flux (million kg/m/day) are overlain on daily moisture flux (thousand kg/m^2^day)) through the vertical column (in blue). Spatiotemporal variability of moisture flux (thousand kg/m^2^day) and integrated moisture flux (million kg/m/day). The plots represent the average over 30 years (1990 to 2019) obtained from ERA5 data. [Figures created using Python script and composited with Microsoft Publisher 365 V2207].

To examine if the amount of atmospheric moisture that can be captured is sufficient for an appropriate infrastructure-based solution, we examine the amount of moisture flux that has been historically available at various locations around the globe. We use ERA5 data over a 30-year period from the year 1990 to 2019, which is available for model grids of size 0.25° × 0.25°. We use the grid points that are completely over the coastal environments but closest to land masses to compute the volume of moisture flux as a function of altitude, as we see in Figure S1 (See Methods section for details of the computation of moisture flux). We assume that any infrastructure that is designed for capturing the humid air will necessarily be placed at a certain height above the ocean surface to protect it from variations in sea level and waves. Therefore, we ignore the first 10 meters above the surface and compute the daily moisture flux as a function of altitude. We illustrate the daily and monthly variation through a 100 m tall (10 m to 110 m above sea level) atmospheric sub-layer for 14 selected locations as shown in Fig. 2. These locations, selected to represent the climatic variations, are close to high population centers that are near oceans across water-stressed regions around the globe. As expected, they are in the subtropical regions of both the northern and southern hemispheres, where the largest arid and semiarid areas exist. The location of the sites is available in Table S1. The average specific humidity near the selected cities ranges between 9 and 20 g/m$^3$, while the mean annual temperature ranges between 14 °C and 30 °C. Many of the sites selected have moderate to high air temperature and medium to high humidity levels.

Figure 2 shows the moisture flux along the vertical, and the mean monthly integrated moisture flux for the 100 m tall surface sublayer of the atmosphere using the 30 years of ERA-5 data. In general, at the daily time scale, the moisture flux increases slightly with altitude across all locations, consistent with the explanation provided earlier. The monthly average is higher during the summer months as should be expected and provides the best opportunity for capturing moisture, with 30 year average in the northern hemisphere ranging between 0.60 and 1.45 million liters/m/day. In general, the four summer months (June-September) can provide between 40% to 55% of the yearly total integrated moisture flux in the northern hemisphere. Among these locations, the largest peak was observed near Chennai in India, in the Bay of Bengal, owing to the monsoon effect. Also, the minimum integrated moisture flux was observed as low as 0.3 million liters/m/day in the winter months in the Tyrrhenian Sea near Rome in Italy. The critical takeaway is that the amount of potential water available for capture has a seasonal variability due to variations in solar radiation, temperature, and other meteorological conditions. Water availability is maximized during the warmer periods of the year when human water demand is also the highest.

We note that the annual potential water yield across all the locations is of the same order of magnitude, even though there is a range of spatial and temporal variability in the moisture flux across these locations. Seasonal variation in the water yield is not a cause for concern. While seasonal variability of the moisture flux is evident in Fig. 2, even the lowest supply of the moisture flux across the seasons has the potential to produce enough water to sustain a sizable population. Storage capacity created through reservoirs can be an effective way to mute the seasonality and provide consistent yield for usage. The finding illustrates that coastal regions with higher water stress align with greater potential for addressing the problem by capturing moisture from proximal oceanic atmosphere environments. For an average consumption rate of 300 liters/capita/day^25^ we see that the amount of water yield by a single 210 m wide and 100 m tall facility can meet the needs of 0.34–0.69 million people across the selected sites with an average of about 0.5 million people. We also see that the entire potable needs of the existing population in these coastal communities can be met by a handful of appropriately engineered structures (Table 1). The annual water yield ranging from a low of about 37 billion liters to a high of over 78 billion liters is sufficient to provide for the needs of the near-coastal population centers with less than ten facilities, with Karachi in Pakistan being an exception due to its extremely large population. We note that the water produced can also be used for non-potable use, such as to meet agricultural or industrial needs. We use potable water only to offer a meaningful interpretation of the potential volume of water available. We also surmise that if this water is used in conjunction with already existing sources, it can augment the freshwater supply for a significantly larger population.

**Table 1:** Assessment of the volume of annual water yield from a facility of dimension 210 m in width and 100 m in height placed closest to large cities in water-stressed zones and the number of people it can serve to meet their entire need estimated at 300 liters (*l*) per capita per day.

Ocean / Sea /Gulf	Country	City	Annual Potential Water Yield (Billion *l* / *y***)**	No. of people served (million) at 300 *l* / *c* / *d*	City Population (million)	No. of facilities to fully serve the city population
Persian Gulf	UAE	Abu Dhabi	54.4	0.50	1.45	3
Balearic Sea	Spain	Barcelona	40.4	0.37	1.62	5
Bay of Bengal	India	Chennai	78.3	0.71	7.09	10
Persian Gulf	Qatar	Doha	55.0	0.50	2.38	5
Indian ocean	South Africa	Durban	71.7	0.65	5.95	10
Arabian Sea	Pakistan	Karachi	74.8	0.68	14.91	22
North Atlantic Ocean	Portugal	Lisbon	49.2	0.45	0.51	2
North Pacific Ocean	USA	Los Angeles	49.7	0.45	3.97	9
Red Sea	Saudi Arabia	Mecca	75.1	0.69	1.58	3
Indian ocean	Australia	Perth	59.4	0.54	1.99	4
Tyrrhenian Sea	Italy	Rome	37.6	0.34	2.87	9
Eastern Mediterranean Sea	Israel	Tel-Aviv	45.7	0.42	0.44	2
Western Mediterranean Sea	Libya	Tripoli	46.1	0.42	3.07	8
South Pacific Ocean	Chile	Valparaíso	45.2	0.41	2.95	8

**Figure 3:**
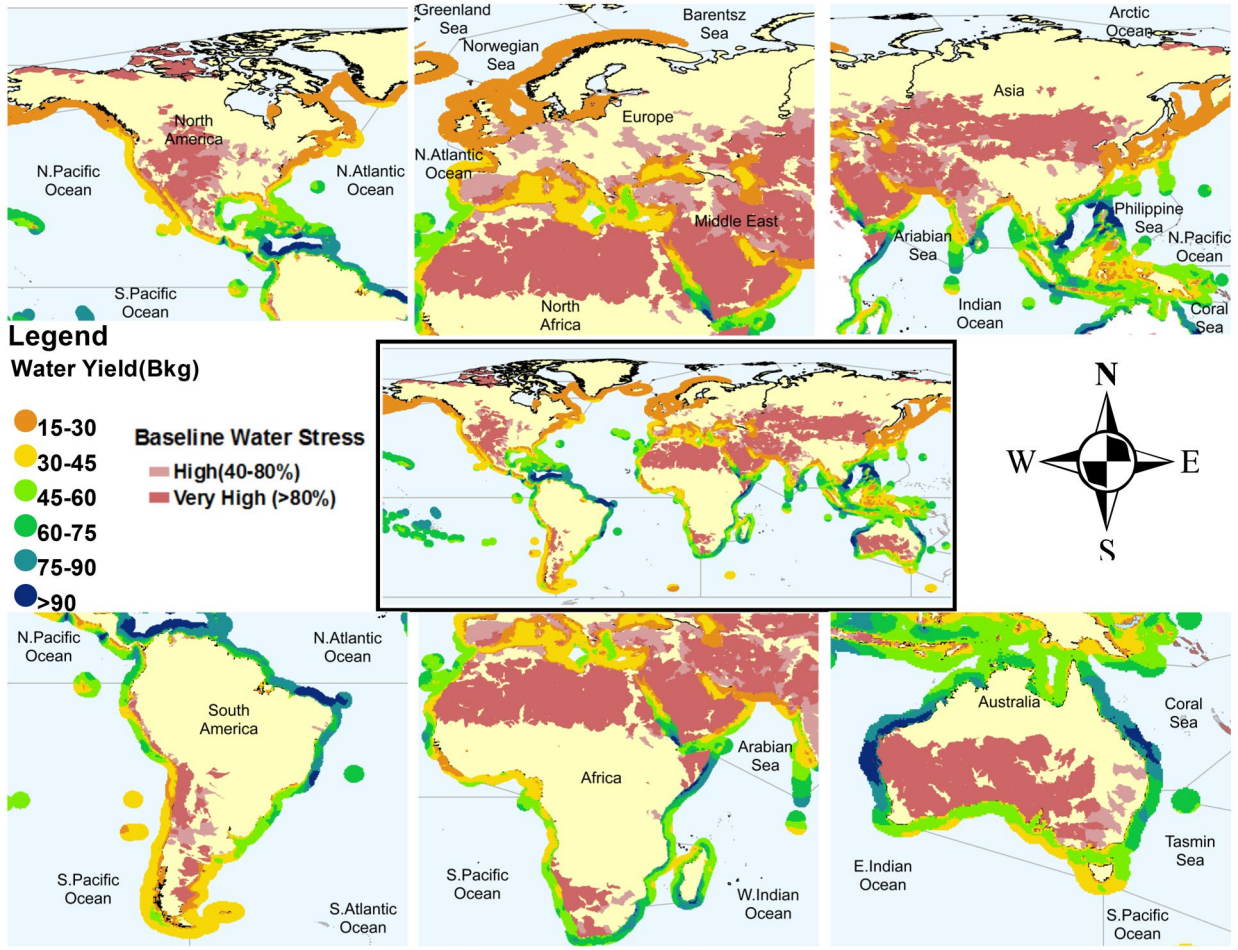
Spatial variability of water yield along the delineated near-offshore region of 200 km across the world. The colors represent the annual potential water yield in billion kgs.The output stands for a hypothetical intake of 100 m in height and 210 m in width. [Figures created using Python script and composited with Microsoft Publisher 365 V2207].

To go beyond the 14 selected locations used for illustrating the feasibility of the proposed approach, we delineated a swath of 200 km over the oceans adjacent to the land along the world’s coasts. We compared the annual potential water yield from a 210 m wide and 100 m tall surface sublayer of atmosphere in a similar manner. The zones for higher water yield from a thirty-year average along the continents are shown in Fig. 3. For a major part of Asia, Europe, and North America, we can obtain an annual water yield of around 10 billion liters. The northern part of South America, Eastern South Africa, and Northeastern Australia can provide an annual water yield higher than 60 billion liters. These results are important because they demonstrate that there is a significant potential to obtain freshwater across the oceans proximal to the coasts of water-stressed regions. The water can also be transported significant distances inland to meet or augment critical needs. As a result, such infrastructure need not be located close to population centers, and their placement can be determined through other meaningful societal objectives.

### Impact of climate change on water yield

**Figure 4:**
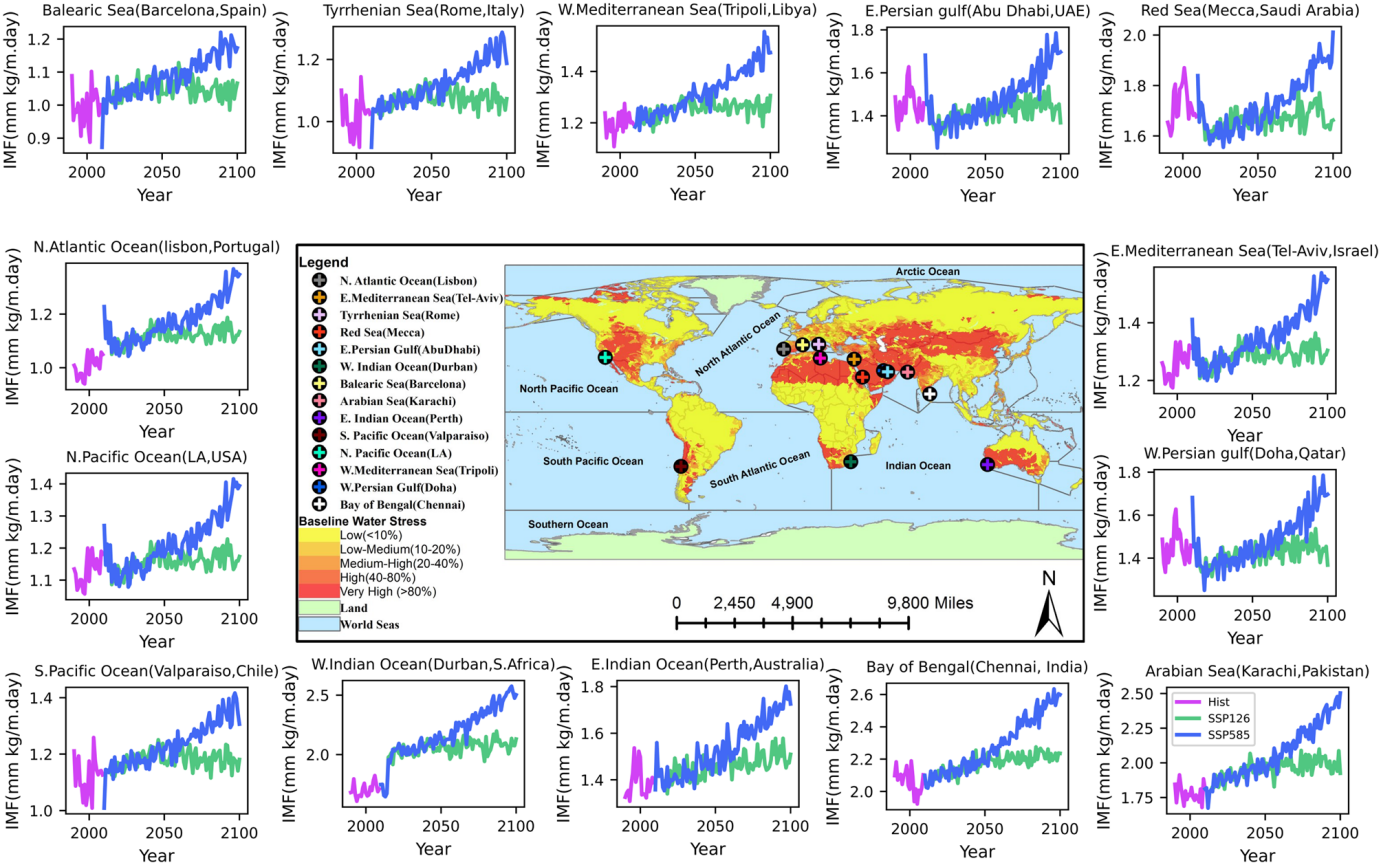
Projection of integrated moisture flux at 14 selected sites obtained from CESM2 WACCM model output. Integrated moisture flux value is in a million kg per day per m width of an atmospheric column from 10 m to 110 m above the sea level. The pink line indicates the historical estimate of yearly mean integrated moisture flux from 1990 to 2019 using the ERA5 data; the blue line is the integrated moisture flux for the SSP585 scenario from 2020 to 2100, and the green lines are the projection of integrated moisture flux for the SSP126 scenario from 2020 to 2100. The latter two are obtained using CESM2-WACCM global circulation model (GCM) data. Due to the mismatch between the spatial resolution of the two datasets, some locations exhibit rapid transitions. [Figures created using Python script and composited with Microsoft Publisher 365 V2207].

To ensure that the feasibility established here, based on the historical data, remains valid for a future changing climate, we examine the trend of moisture flux under two climate change scenarios as shown in Fig. 4. We consider scenario SSP126, which is the most optimistic scenario, and scenario SSP585, which represents the upper bound for the range of warming scenarios in the literature. We see that in both the SSP126 and SSP585 scenarios, the annual mean integrated moisture flux until the year 2100 at all the locations does not decrease. For the SSP585 scenario, it increases everywhere, whereas, for the SSP126 scenario, it remains flat for the Persian gulf near Doha and Abu Dhabi and the Red sea near Mecca. Based on this analysis, we conclude that the water yield from the atmosphere is not likely to decrease, and the future moisture flux trajectory will possibly be in between the trajectory obtained for the SSP126 and SSP585 scenarios, depending upon the climate change realized.

### Thermodynamic and wind effect on moisture flux under climate change

We determine the percentage change in the mean integrated moisture flux for two periods corresponding to 2020 to 2059 and 2060 to 2099 to compare it with the average of 1990 to 2019 for both the SSP126 and SSP585 scenarios for all 14 selected sites (as we see in Figure S2 in the supplementary material). On average, in the SSP585 scenario, the integrated moisture flux increases by around 4% and 16% during 2020 to 2059 and 2060 to 2099, respectively. Maximum percentage increase occurs in the western Indian Ocean near Durban in South Africa, and the minimum percentage increase occurs in the Red Sea near Mecca. We analyzed the corresponding percentage increase in the near-surface specific humidity and wind speed to further investigate how the integrated moisture flux might respond to the changing climate. The average increase in the near-surface specific humidity of the selected sites is 9% and 25% during 2020 to 2059 and 2060 to 2099 in the SSP585 scenario. We observed a consistent increasing pattern in specific humidity at all the locations. Increased sea surface and atmospheric temperature due to global warming result in increased water vapor in the atmosphere, resulting in increased humidity. Indeed, when the atmosphere warms, saturation vapor pressure increases following the Clausius-Clapeyron equation. Over the ocean, this results in more moisture in the atmosphere. On the other hand, the percentage changes of mean surface wind speed show an opposite trend. On average, the wind speed decreases by around 5% during both 2020 to 2059 and 2060 to 2099 in the SSP126 scenarios, and by 5% and 7% during 2020 to 2059 and 2060 to 2099 in the SSP585 scenarios, respectively. The projected increase in near-surface humidity level and the decrease in the predicted wind field suggests that the projected change in integrated moisture flux is dominated by thermodynamics and is not wind-driven. The findings contribute to our assessment that the water yield from oceanic evaporation would be a sustainable approach even under the future climate.

### Upwind fetch and flux footprint over oceans

To assess the source region of oceanic evaporation, we computed the upwind fetch and 2-D footprint that contributes to the moisture flux supply at each of the selected locations. The fetch and the footprint area vary due to wind speed, direction, and atmospheric stability^26^. The spatial extent of the upwind fetch corresponding to 90% contribution of moisture flux ranged mostly from 20 to 40 km across the sites, depicting the same order of magnitude as shown in Figures S3 and S4. Upwind fetch increases non-linearly as a function of moisture flux. For 50% to 70% contribution of flux, the upwind fetch statistics are below 20 km across all the selected sites (Figure S4). The footprint area is well below 500 km^2^ (Figure S5). Persian Gulf near Doha, Qatar, and Abu Dhabi, UAE experience a greater upwind fetch and flux footprint area. These findings depict that the spatial scale of water vapor capture is small compared to the scale of the regional water balance. This analysis indicates that the areal extent of the ocean that contributes to the water yield is relatively small and will not impact the moisture availability in the atmosphere in the context of regional and global circulation.

Approximately $5.21\times 10^{17}$ liters of water evaporate from the earth’s surface (including ocean surface, inland water bodies, soil, and plants) annually^27^. Across all 14 locations, the total amount of water vapor extracted annually by the proposed designed system will be around $7.8\times 10^{11}$ liters, which is approximately 0.0015 % of the total globally evaporated water. Further, we note that most precipitation events are a result of moisture drawn from vast areas that are substantially distant from the precipitation location. For example, atmospheric rivers, monsoon systems, and mesoscale convective systems all have source regions that are orders of magnitude larger than that associated with the fetch of the moisture capture system proposed here and rely on moisture in the entire boundary layer column that is vertically larger by an order of magnitude in comparison to the height of the structure envisioned here. This argument further supports the conclusion that the proposed approach for moisture capture will not have an impact on atmospheric circulation and downwind precipitation patterns.

### Financial feasibility

Having established that there is and will be sufficient moisture in the atmosphere column above oceans near the coastal regions, particularly surrounding the water-stressed regions of the globe, we now address the question of the financial feasibility of the construction of such an infrastructure. Since no such structures exist, we do not ask what it will cost to construct such an infrastructure, but what cost model will make building and operating it feasible. In other words, can we characterize the upper bound of cost structure to be competitive with the current technologies assuming that the marginal cost of water doesn’t increase? We assume that the cost of building a facility for collection and condensation of atmospheric vapor is U.S. $600 million, approximately that of building a large cruise ship or an oil rig. We further assume that the facility is amortized over a 30-year period with a current interest rate of 3.75%. We also assume a present-day operational cost of $175,000 per day with an inflation rate of 2.06%, the average in the USA over the past 20 years. Assuming that 500,000 people benefit from such a facility, the annual cost to build is 67 dollars per beneficiary and the annual cost to both build and operate the facility is $241 per beneficiary (See Table S2 in the supplementary material and the associated Excel file in the online supplementary material for the details of the calculation). In terms of the volume of water, this results in an annual cost of $2.20 per 1000 liters of water which is competitive with the production cost of the desalination plants. However, even after several decades of technological improvement, desalination plants have environmental effects associated with waste disposal. The desalination process produces a residual that is significantly warmer and saltier than that of the input. This residual is released into the sea, where it increases the salinity level and becomes detrimental to the marine ecology^28,29^. In contrast, we believe that the process outlined here to capture water vapor that evaporates naturally over the ocean, transport and condense it to produce liquid water will have no significant environmental impacts in terms of byproduct generation.

## Conclusions

Through this research, we have established that the capture of moisture over ocean surfaces is a feasible solution for many water-stressed regions of the world. The estimated water yield of the proposed structures could alleviate the freshwater needs of large population centers in the subtropics. The average and range of the water yield establish the feasibility of the proposed approach to address water security, both under existing and future climate. This proposed concept could be used as a substitute or to supplement the year-round freshwater production in areas with access to coastal water bodies or transported to distant inland locations, thereby assisting in alleviating water scarcity while also maintaining ecosystems and the environment. We note that the proposed concept of utilizing atmospheric humidity for potable water production is notably different from previous articulations which include water production over land by radiative cooling^30–38^, active cooling by vapor compression refrigeration cycle^39–48^ or thermoelectric cooling^49–53^ and desiccant method^40,54–65^. These alternate solutions are not scalable to address water scarcity concern in a significant way because the amount of moisture flux available in the atmosphere over land is substantially smaller than over the large oceanic sources. Where feasible, small islands in the oceans could also serve as sites for our proposed facilities, potentially resulting in reduced cost, provided that humidity and wind fields are primarily determined by the surrounding water body and not the landmass. The proposed solution is scalable, has negligible environmental costs, and increases in capacity under warmer climate conditions. Our estimates of water yield are based on assuming that all moisture carried by the ambient wind can be extracted. We believe that if suction/compressor is used, then loss in efficiency during the intake and transport process can be overcome. We also believe that the energy cost of this endeavor will not be burdensome as moisture that is captured is already evaporated by the solar energy, and efficient approaches can be deployed for achieving efficient condensation processes.

## Methods

### Data

We used ERA-5 daily data with a resolution of $0.25^{\circ } \times 0.25^{\circ }$ over oceans due to its agreement with a range of observed measurements^66,67^. We use surface data for 1990 to 2019 at 10 m elevation for wind speed and at 2 m elevation for air temperature, dew point temperature, instantaneous vapor flux, surface sensible heat flux, friction velocity, and surface air pressure. According to the sign convention of ERA-5, vertical downward fluxes are positive. Data on specific humidity are not readily available from ERA-5 data on single levels, and therefore we estimated the daily 2-m specific humidity from dew point temperature and surface air pressure using the moist thermodynamics formulation^68^. The saturation vapor pressure computed from the dew point temperature in the Clausius-Clapeyron equation represents the actual vapor pressure as shown in Eq. (1)^69^. We get the 2 m-specific humidity from the dependence between the actual vapor pressure and the specific humidity as shown in Eq. (2)^69^.

1 $$\begin{aligned} e= 611 exp \left[ \frac{L_v}{R_v}\left( \frac{1}{273.15}-\frac{1}{T_d}\right) \right] \end{aligned}$$

2 $$\begin{aligned} q= & {} \frac{0.622 e}{P_a-0.378e} \end{aligned}$$

Here, *e* is the actual vapor pressure at temperature T; $L_v$ is the latent heat of vaporization; $T_d$ is the dew point temperature; $R_v$ is the specific gas constant for water vapor (461.5 J/kg/K); *q* is the specific humidity at 2 m; and $P_a$ is the surface air pressure at 2 m. For the estimation of moisture flux under climate change scenarios, we used data from the CESM2-WACCM GCM model with ensemble member r1i1p1 with a horizontal resolution of 1$^{\circ } \times$1$^{\circ }$ from the CMIP6. CESM2 is chosen because it contains an improved representation of the teleconnections with ENSO and Madden-Julian Oscillation, reduced shortwave cloud forcing biases, and greater climate sensitivity. Also, CESM2 possesses better agreement with the observed trend of global land carbon accumulation^70^. WACCM has been selected because this dataset contains the required variables for calculating moisture flux. SSP126 (combining SSP1 and RCP2.6) and SSP585 (combining SSP5 and RCP8.5) are chosen as climate change scenarios to compute the moisture flux and potential freshwater yield for the future. SSP126 represents both an optimistic global warming and minimal mitigation challenges, whereas SSP585 represents the same for the pessimistic scenario^71^.

### Governing equations for moisture flux estimation

Moisture flux is defined as water vapor passing through a unit vertical area per unit time. The flux transported by the mean wind contributes to the mean moisture flux, and the flux transported by the eddies contributes to the turbulent component of moisture flux. Mean horizontal wind primarily dominates the advective transport of humidity, and therefore, we have considered the mean advective moisture flux and ignored the turbulent component. Moisture flux is obtained as the mean of the product of the air density ($\rho$), specific humidity (*q*), and wind speed (*u*), as shown in Eq. (3). We divided the 100m vertical column into 10 m tall strips and summed up the moisture flux ($m_i$) for each strip (*i*) to get the mean integrated moisture flux (*IMF*) for the layer height as shown in Eq. (4). We assume that the moisture flux computed for a unit width can be simply scaled for smaller widths as there is no data to capture horizontal variation within the climate model resolutions:

3 $$\begin{aligned} m_i=   \rho _a \overline{ q_iU_i} \end{aligned}$$

4 $$\begin{aligned} IMF=  \sum _{i=1}^{n}\rho _a  \overline{ q_iU_i} \end{aligned}$$

5 $$\begin{aligned} APWY= & {} IMF\times w\times 24\times 3600\times 365 \end{aligned}$$

Here, $\overline{ q_iU_i} = \overline{ q_i}\overline{U_i} + \overline{q_i^{'} U_i^{'}}$. Here $\overline{ q_i}\overline{U_i}$ and $\overline{q_i^{'}U_i^{'}}$ are the mean and turbulent component of kinematic moisture flux. $m_i$ is the moisture flux in kg of water/m^2^s for the $i^{th}$ layer in the surface sublayer of the atmosphere, $\rho _a$ is the air density specified as 1.12 kg/m^3^, $q_i$ is the specific humidity and $U_i$ is the horizontal wind which is obtained from the zonal (u) and meridional wind (v) components as $U_i=\sqrt{u^2_i+v^2_i}$; w is the width of the intake of the hypothetical water vapor harvesting system. Annual potential water yield (*APWY*) is simply the product of the integrated moisture flux (*IMF*) per unit width, the width of the water vapor collection system (*w*), and the number of seconds in a whole year as we see in Eq. (5).

For the calculation of moisture flux for each strip between heights $z_{j+1}$ and $z_j$, wind speed and specific humidity profiles are obtained from the flux profile relationship invoked from Monin–Obukhov similarity theory^24,72^ as shown in Eqs. (6) and (7), which assumes horizontal homogeneity and zero subsidence:

6 $$\begin{aligned} q_{j+1}= &  q_j-\frac{H}{a_v k u_* \rho }\left[ ln \frac{z_{j+1}-d_0}{z_j-d_0}-\Psi _v\left( \frac{z_{j+1}}{L}\right) +\Psi _v\left( \frac{z_j}{L}\right) \right] \end{aligned}$$

7 $$\begin{aligned} u_{j+1}= &  u_j+\frac{u_*}{k}\left[ ln \frac{z_{j+1}-d_0}{z_j-d_0}-\Psi _m\left( \frac{z_{j+1}}{L}\right) +\Psi _m\left( \frac{z_j}{L}\right) \right] \end{aligned}$$

Since assumptions of horizontal homogeneity and zero subsidence are valid for the atmosphere above large water bodies, moisture flux in the atmosphere above marine water bodies would follow the similarity relations. Here, $u_*$ is the friction velocity, $d_o$ is displacement height (0.001 m), $\Psi _h$, $\Psi _v$ and $\Psi _m$ are the flux profile function for heat, water vapor, and momentum that varies depending on the stability of the atmospheric layer, $a_v$ or $a_h$ is the ratio of eddy diffusivity and eddy viscosity under neutral condition, for water vapor and heat respectively and *k* is the von Kármán constant. Stability of the atmospheric layer is obtained from the Obukhov’s Stability length, *L*^24^, as shown in Eq. (8). Obukhov length is positive for stable and negative for unstable atmospheric stratification and becomes near-infinite in the limit of neutral stratification:

8 $$\begin{aligned} L= &  -\frac{u_*^3 \rho _a}{a_h k\left[ \left( \frac{H}{T_a C_p}\right) +0.61E\right] } \end{aligned}$$

Here, *L* is the stability length in meters, *E* is the instantaneous evaporative flux (kg/m^2^s), *H* is the sensible heat flux (J/m^2^s) and $T_a$ is the atmospheric temperature at 2 m elevation. We calculate the mean daily moisture flux from 1990 to 2019 for each of the selected grids. The regions were extracted using the polygon shapefile from the world’s marine water bodies for the historical and future climate period. For the historical moisture flux analysis, we generate a mean representative annual time series of moisture flux from 30 consecutive years of outputs from 1990 to 2019. Spatially averaging the grids gives a representative daily moisture flux time series for the selected zones. Besides, we compute the spatially averaged integrated moisture flux for historical and future climatic periods for each of the selected regions to compare the moisture flux for the selected areas across the globe. The daily fields were then averaged to monthly and yearly mean values. The specific humidity and wind speed were retrieved at a daily resolution from the selected CMIP6 model to analyze the percentage change in the upcoming decades.

### Fetch and footprint for the estimated moisture flux over oceans

The oceanic surface area that contributes to the flux captured by the intake is termed as the flux footprint, and its maximum extent in the upwind direction is the fetch of the flux over oceans. Both the fetch and footprint change dynamically and increase with the height of the intake structure, atmospheric stability, and wind direction. We estimated the fetch and the flux footprint associated with the top of the intake structure (110 m elevation), which corresponds to the maximum extent. This information helps us estimate how far from the nearest shore we need to go to locate the intake structure to ensure that the land area within the footprint will not reduce the evaporative flux.

Here, we calculate the footprint climatologies using the parameterization of the two-dimensional footprint prediction model developed by Kljun et al.^26^, which considers the impact of zonal and meridional components of wind, surface roughness, and atmospheric stability. Kljun et al. established the parametrization based on the backward Lagrangian stochastic particle dispersion model^26^. This parameterization is valid for a broad range of stability conditions and measurement heights over the entire planetary boundary layer. For an atmosphere above ocean surfaces, surface roughness length is very small. Atmospheric thermal stability for such an environment is greatly affected due to variations in air temperature. The flux footprint model, which assumes stationarity across the integration period (daily resolution) and horizontal homogeneity of the flow, provides the extent of the two-dimensional flux footprint in the upwind and cross-wind directions at any given time.

We used this FFP model as a function on a loop in our Python code to estimate flux footprint climatologies using the ERA-5 dataset for each day over 30 years (1990-2019) for each of the 14 selected sites. Mean monthly statistics of upwind fetch and area of the footprint were then calculated using the output of daily footprint climatologies. While estimating the fetch of the two-dimensional flux footprint, we define a set of contours (r) to delineate the source areas up to a certain percent of flux contribution. We estimate the upwind fetch corresponding to 50%, 70%, and 90% contribution of the moisture flux observed by the capture surface of the intake. We use the upper limit for our study as 90% contribution of the moisture flux because the contribution falls of slowly beyond that point. Since fetch is the distance from the capture surface of the intake, it increases as the percent contribution increases. We used the ‘Fetch rose’ diagram^73^ following the Weibull probability density functions as a graphic tool to give a succinct view of how the fetch for the 90% flux contribution is typically distributed and oriented at the selected sites. It describes the distance and direction that contributes to the moisture flux observed at the intake. We also obtain the seasonal variation of the area of flux footprint, showing the statistics of the dynamic source areas that contribute to 90% of the prevailing flux.

To investigate upwind fetch and flux footprint, this study required meteorological data such as mean wind speed at the top of the intake structure, boundary layer height, Monin-Obukhov length, the standard deviation of lateral velocity fluctuations, friction velocity, wind direction, and the measurement height, which is the maximum elevation of the capture surface in this case. Wind direction has been calculated using the zonal and meridional components of wind data as mentioned in^74^. We adopt a convention that a northerly wind is 0$^{\circ }$. The daily value of the standard deviation of lateral velocity fluctuations was calculated using the hourly data of the meridional component of wind (*v*) from ERA-5. We used the boundary layer height data from the ERA-5. It is calculated from the bulk Richardson number method, which is suitable for convective and stable boundary layer conditions. Equation (8) is used to compute the Monin-Obukhov length that represents the condition of atmospheric stability structure. We kept the value of the displacement height, *d*, as zero as we are dealing with the atmosphere above the ocean surface. The measurement height is calculated as (*z − d*), where *z* = 110 m which is the elevation of the top of the capture surface of the intake. Using the input data to the 2-D flux footprint model by Kljun et al.^26^ provides the contour lines in the Cartesian coordinate system (*x* and *y*) for the particular percentage of the footprint as input. Sci-kit, an image processing library based on the Douglas-Peucker algorithm, has been used to obtain the major and minor axis of the ellipse model created from the contour point of *x* and *y*. The major axis represents the fetch distance in the upwind direction from the installed intake of the water vapor collection system. The area enclosed within the contour points of the footprint is the footprint area obtained from the sci-kit documentation.

## Supplementary Information

Supplementary Information.

## Data Availability

ERA-5 hourly data on single levels have been used for conducting the analyses presented in this study. The data is publicly available here:http://bit.ly/3ENBxbT.
