# Supplementary material for: Increasing freshwater supply to sustainably address global water security at scale
Source: Sci Rep. 2022 Dec 6;12:20262. doi: 10.1038/s41598-022-24314-2 (PMC9726751; doi:10.1038/s41598-022-24314-2)
Supplement: Supplementary file 1 — Supplementary Information. [file 41598_2022_24314_MOESM1_ESM.pdf]

Increasing Freshwater Supply to Sustainably Address Global Water Security at Scale: Online Supplementary Information

Afeefa Rahman,[1] Praveen Kumar,[1,2,*] Francina Dominguez[2]

[1]Department of Civil and Environmental Engineering,University of Illinois at Urbana-Champaign;
Illinois, USA
[2]Department of Atmospheric Sciences,University of Illinois at Urbana-Champaign;
Illinois,USA

*Corresponding author: Praveen Kumar, e-mail: kumar1@illinois.edu;

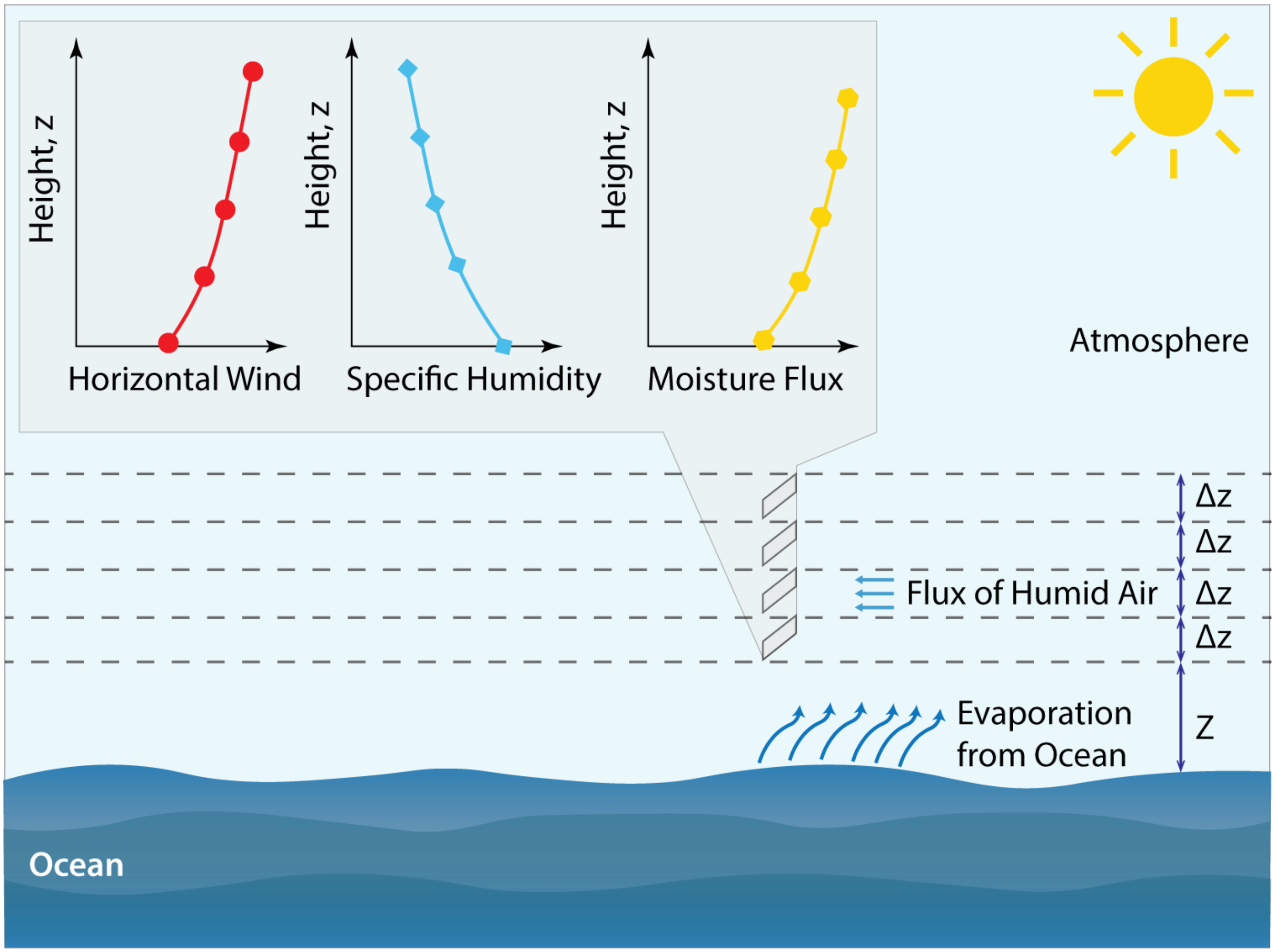

Figure S1. As we move above the ocean surface, the wind speed (U) increases while the humidity (q) decreases. The moisture flux in the atmosphere is a function of wind speed and specific humidity. The latter implicitly depends on the vertical temperature profile. The net result is that the moisture flux $MF = \rho_a q U$.Where ρ_a= 1.12 is the density of moist air, increases with altitude due to a stronger wind effect. The integrated moisture flux through an atmospheric column is approximated as the sum of the fluxes through computational layers of vertical thickness Δz and horizontal width of $1m$ orthogonal to the wind direction. [Figures created using Python script and composited with Microsoft Publisher 365 V2207.]

Table S1. Average Meteorological Conditions at the Chosen Study Locations

Location			Avg. Temp. (C)	Abs. Humidity (g/m^3)	Avg. Wind (m/s)	Lat, Lon	Water Stress Rank	Population (million)
**Ocean / Sea / Gulf**	**Country**	**City**						
Persian Gulf	UAE	Abu Dhabi	27.53	17.3	4.71	25.38,53.00	10	1.45
Balearic Sea	Spain	Barcelona	17.82	10.46	5.82	40.27,3.75	28	1.62
Bay of Bengal	India	Chennai	27.98	19.78	5.48	11.15,81.55	13	7.09
Persian Gulf	Qatar	Doha	27.53	17.3	4.71	25.38,53.00	1	2.38
W.Indian ocean	South Africa	Durban	22.59	13.63	7.3	-29.36,33.75	48	5.95
Arabian Sea	Pakistan	Karachi	26.35	17.38	5.55	23.53, 65.85	14	14.91
N. Atlantic Ocean	Portugal	Lisbon	16.88	10.02	6.93	38.42,-11.68	41	0.52
N. Pacific Ocean	USA	Los Angeles	14.57	9.23	7.49	33.58,-122.19	71	3.97
Red Sea	Saudi Arabia	Mecca	29.03	19.61	5.42	18.58,39.62	8	1.58
E.Indian ocean	Australia	Perth	18.54	10.01	8.18	-32.10,114.19	50	1.99
Tyrrhenian Sea	Italy	Rome	17.97	10.64	5.27	40.83,11.71	44	2.87
E. Mediterranean Sea	Israel	Tel-Aviv	20.89	12.33	5.38	33.10,32.56	2	0.43
W. Mediterranean Sea	Libya	Tripoli	20.2	11.89	5.78	34.23,14.29	6	3.07
S. Pacific Ocean	Chile	Valparaíso	14.39	8.96	7.03	-32.98,-73.35	18	2.95

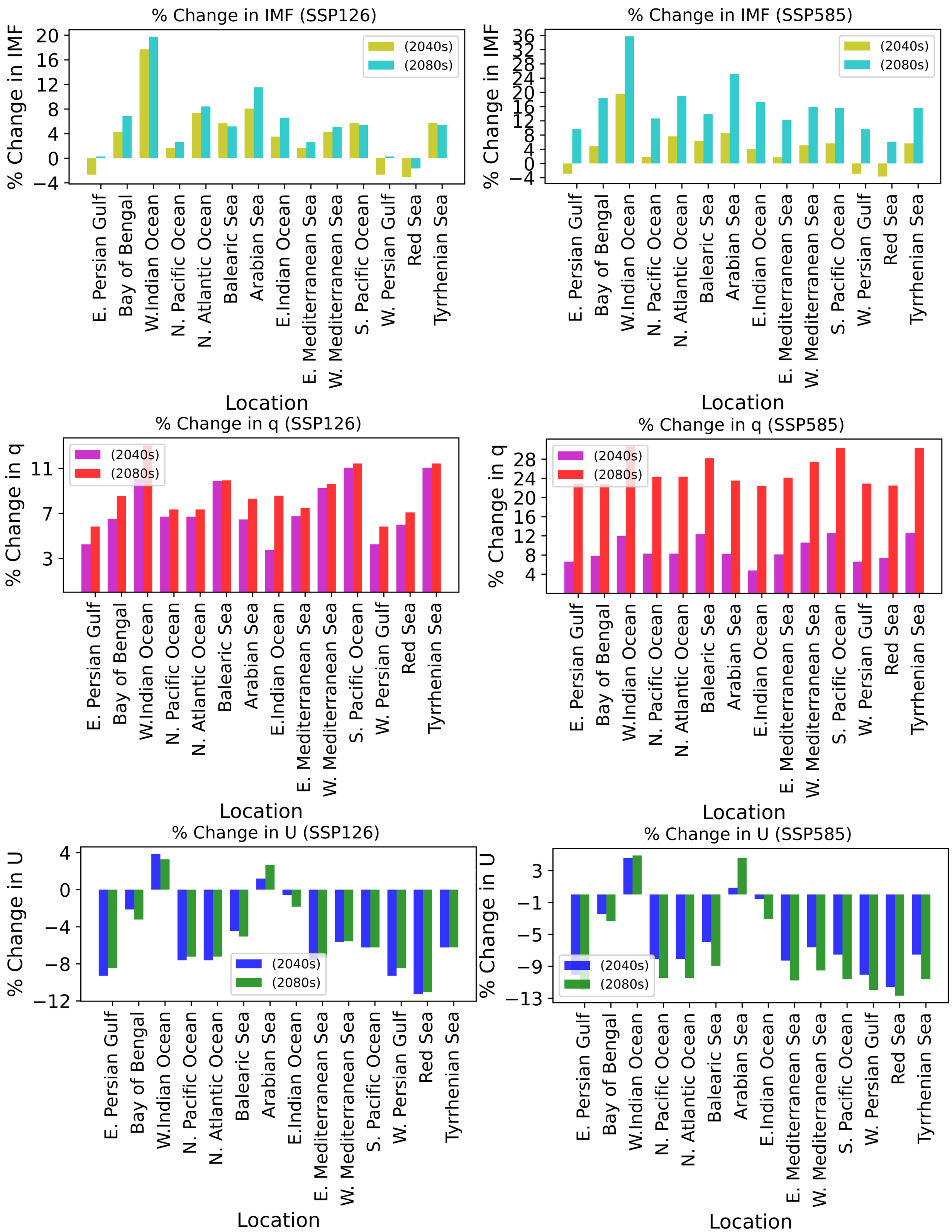

Figure S2. Percentage change in the integrated moisture flux (IMF), specific humidity, and horizontal wind speed at 14 locations for SSP126 and SSP585 scenarios. The 2040s and 2080s mean a 30-year average from 2020 to 2059 and from 2060 to 2099, respectively. Integrated moisture flux is the daily flow rate of water vapor through a column of 100 m in height per meter orthogonal to the wind flow. U is the mean horizontal wind speed, and q is the specific humidity in kg of water vapor per kg of moist air. For both scenarios, the percentage increase in specific humidity is larger than that of wind speed. Analysis of near-surface specific humidity and wind field shows that the expected pattern of moisture flux change is dominated by thermodynamics and not by wind. [Figures created using Python script and composited with Microsoft Publisher 365 V2207.]

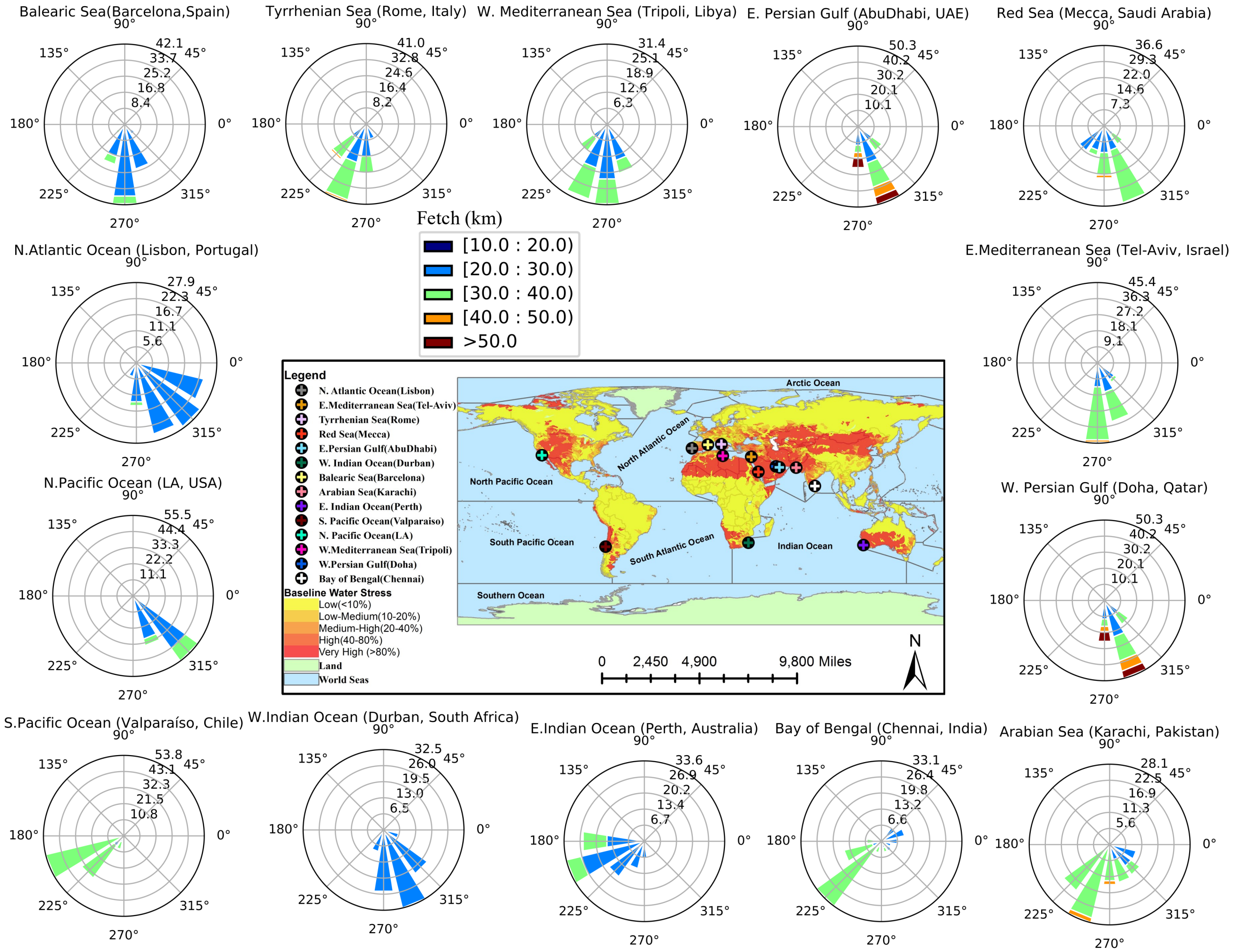

Figure S3. Fetch rose diagram for 90% contribution of flux observed at each of the 14 selected locations. The diagram represents the mean daily upwind fetch for a whole year calculated from 30 years (1990 to 2019) of input data obtained from ERA-5. The range of the colors represent upwind fetch in kilometers. The circular format of the fetch rose shows the upwind direction. How often the fetch is observed from a particular direction in the 30-year data is indicated by the length of each "spoke" around the circle. For most of the locations, the observed fetch is within the range of 20-40 *kms*, with the south-west and south-east being the predominant direction of the upwind fetch. [Figures created using Python script and composited with Microsoft Publisher 365 V2207.]

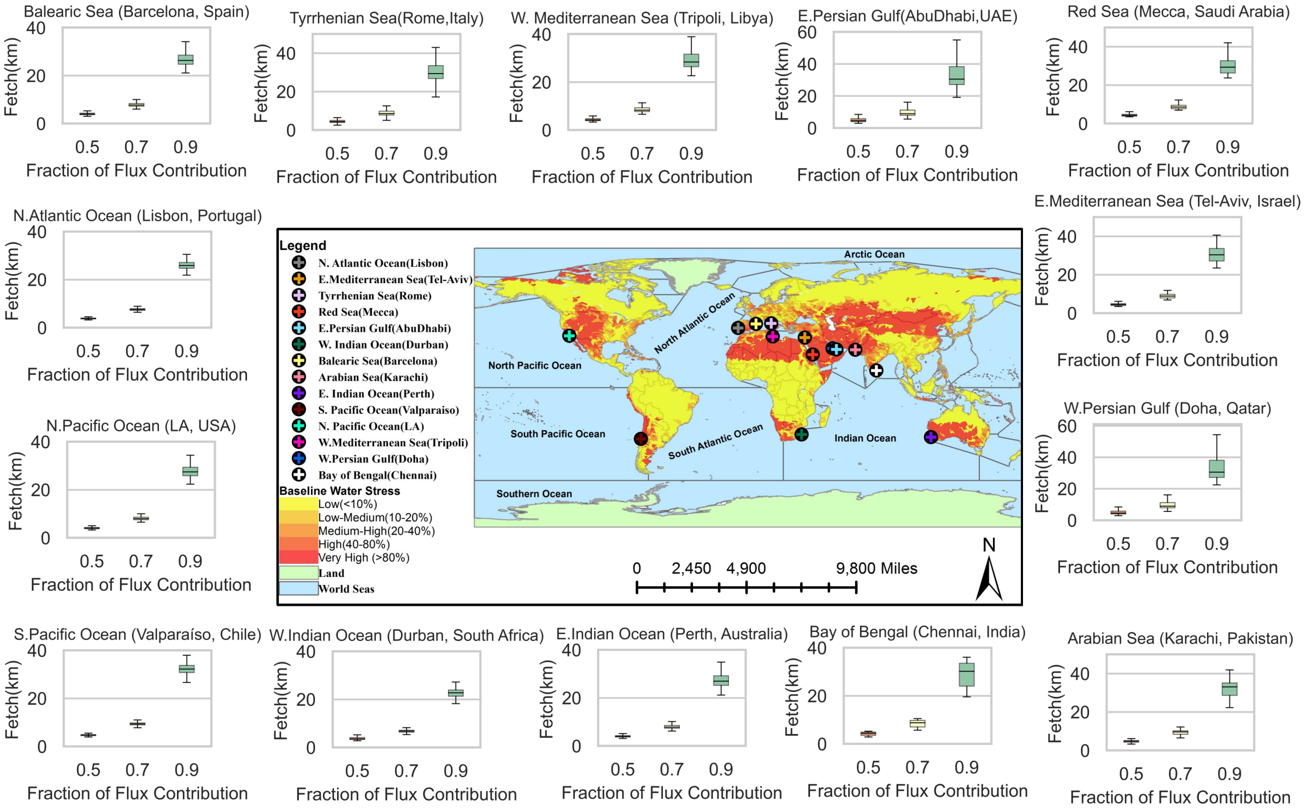

Figure S4. Comparison of the mean representative upwind fetches observed at the intake for 50%,70% and 90% contribution of the flux. For all the locations, at least 70% of the prevailing flux can be captured from within a distance well below 20 *kms*. The upwind direction for all locations is shown in Figure S4. The fetch distance indicates the distance from the nearest shoreline for the intake structure to ensure maximum moisture availability. [Figures created using Python script and composited with Microsoft Publisher 365 V2207.]

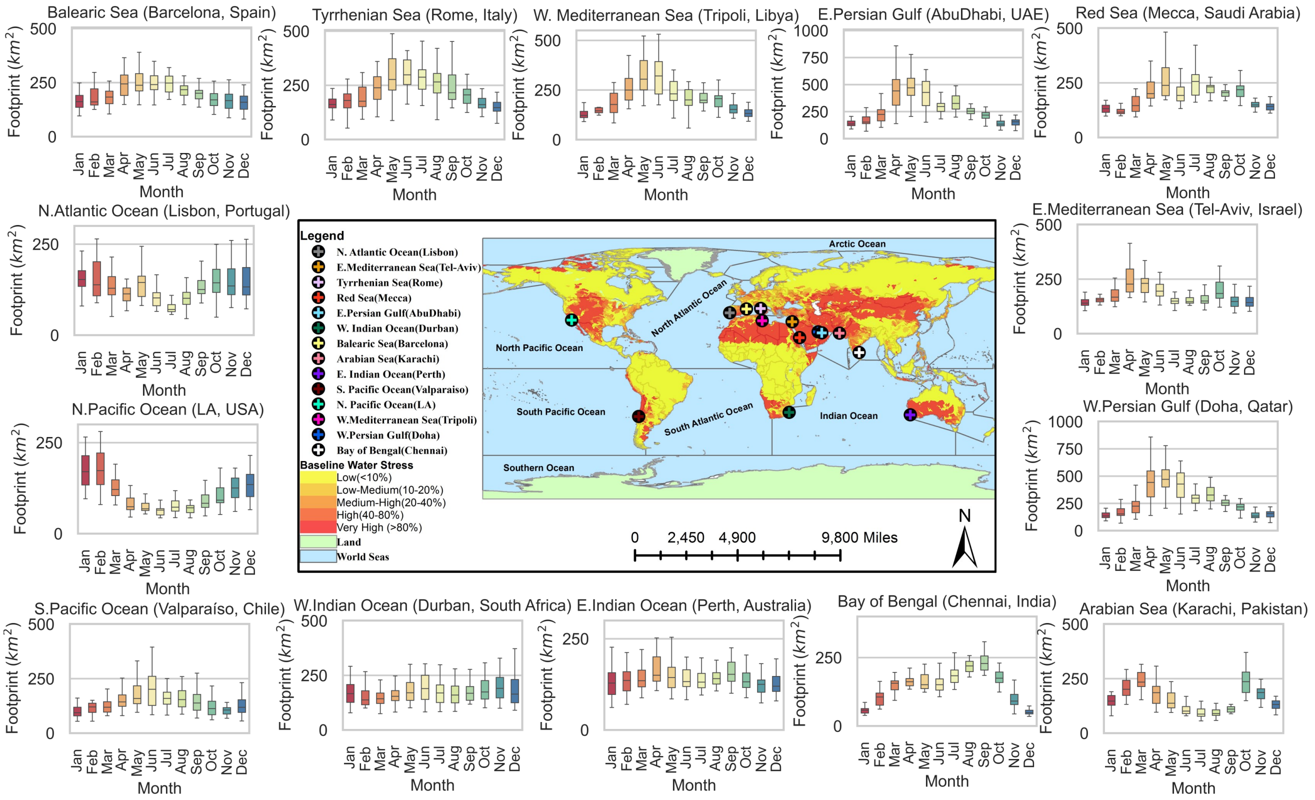

Figure S5. The statistics of flux footprint for each month of the year that contributes to the supply of the flux, calculated from 30 years of data. Seasonal fluctuation in the flux footprint varies across the sites. The observed footprint area is well below 500 km^2 for all the study sites except the Persian Gulf. Persian gulf experiences the highest range, with a peak above 750 km^2. [Figures created using Python script and composited with Microsoft Publisher 365 V2207.]

Table S2. Hypothetical cost structure to build and operate a facility to extract fresh water from water vapor in the atmosphere above the ocean (The Excel file is also made available as online supplementary material for the details of the calculation).

**Cost Category**			**Cost**	**Gallons**	**Liters**
	Expected number of people benefitting per facility	500000			
	Volume of water needed per person per day			79	300.0
	Total volume of extracted water to meet total needs of each beneficiary			39,625,827	150,000,000
**Fixed cost**	Cost (assume $500M for water and $100M for power)		$600,000,000		
	Amortization period or duration of useful life (yrs)	30			
	Interest rate	3.750%			
	Cost for the total amortization period		$1,000,329,678		
	Annual cost (total / duration)		$33,344,323		
	**Annual cost to build (per beneficiary)**		**$67**		
**Operational & Maintenance Cost**					
	O&M Cost per day in year 1 (assume)	$175,000			
	Annual O&M Cost in year 1		$63,875,000		
	Inflation rate	2.06%			
	Total O&M cost over project life factoring for inflation		$2,615,777,173		
	**Average annual O&M cost per beneficiary over project life**		**$174**		
	**Total annual cost to build and operate per beneficiary (present value)**		**$241**		
	**Cost per day per beneficiary (present value)**		**$0.66**		
	**O&M as a Percentage of total cost**		**78.33%**		
**In terms of volume of water**					
**Cost to build**	Cost per gallon (liter) of water			$0.00231	$0.00061
	Cost per 1000 gallons (liters)			$2.31	$0.61
	Cost per million gallons (liters)			$2,305.42	$609.03
**Cost to build and operate**					
	Cost per gallon (liter) of water			$0.00833	$0.00220
	Cost per 1000 gallons (liters)			$8.33	$2.20
	Cost per million gallons (liters)			$8,333.91	$2,201.59
